# Modulation of Oxidative Stress: Pharmaceutical and Pharmacological Aspects 2017

**DOI:** 10.1155/2017/4802824

**Published:** 2017-12-17

**Authors:** Luciano Saso, Liudmila Korkina, Neven Zarkovic

**Affiliations:** ^1^Department of Physiology and Pharmacology “Vittorio Erspamer”, Sapienza University of Rome, Rome, Italy; ^2^Centre for Innovative Biotechnological Research, Moscow, Russia; ^3^Rudjer Boskovic Institute, Zagreb, Croatia

Reactive oxygen and nitrogen species are generated during normal cellular metabolic activities but can also play an etiopathogenetic role in a variety of conditions, including cardiovascular diseases, neurodegenerative diseases, and cancer.

Although the role of oxidative stress in human physiology and pathology has been intensely studied for several decades, it is still far from being understood. Certainly, the roles of free radicals and antioxidants have been significantly redefined. Some “negative” actions of free radicals in human biology and pathology are now known to be “positive,” and the hypothesis that “classical antioxidants” (radical scavengers) could be always beneficial for the human health was not confirmed by several epidemiological and clinical studies. The possible reasons of the failures of the current antioxidant therapies, including methodological pitfalls in the drug development and delivery and the lack of good biological markers to select the patients, have been reviewed elsewhere [1, 2].

We currently believe that instead of “antioxidants,” it is more appropriate to develop “modulators of oxidative stress” because, depending on the condition, it could be more beneficial to reduce or increase the oxidative stress.

In this special issue, several aspects of the modulation of oxidative stress were examined including the role of polyphenols (“Cytoprotective Mechanisms Mediated by Polyphenols from Chilean Native Berries against Free Radical-Induced Damage on AGS Cells” by Á. Felipe et al., “Curcumin Protects Skin against UVB-Induced Cytotoxicity via the Keap1-Nrf2 Pathway: The Use of a Microemulsion Delivery System” by M. B. Y. Greenwald et al., “Synthetic Isoliquiritigenin Inhibits Human Tongue Squamous Carcinoma Cells through Its Antioxidant Mechanism” by C. Hou et al., and “Oxidative Stress Triggered by Apigenin Induces Apoptosis in a Comprehensive Panel of Human Cervical Cancer-Derived Cell Lines” by R. P. Souza et al.) and other natural substances (“A Clinically Relevant Variant of the Human Hydrogen Sulfide-Synthesizing Enzyme Cystathionine *β*-Synthase: Increased CO Reactivity as a Novel Molecular Mechanism of Pathogenicity?” by J. B. Vicente et al., “Protective Mechanisms of the Mitochondrial-Derived Peptide Humanin in Oxidative and Endoplasmic Reticulum Stress in RPE Cells” by L. Minasyan et al., “Effect of Emodin on Preventing Postoperative Intra-Abdominal Adhesion Formation” by G. Wei et al., and “*Opuntia* spp.: Characterization and Benefits in Chronic Diseases” by M. del Socorro Santos Díaz et al.), some cardiovascular effects of modulators of oxidative stress (“Pentaerythritol Tetranitrate In Vivo Treatment Improves Oxidative Stress and Vascular Dysfunction by Suppression of Endothelin-1 Signaling in Monocrotaline-Induced Pulmonary Hypertension” by S. Steven et al., “4-Hydroxynonenal Contributes to Angiogenesis through a Redox-Dependent Sphingolipid Pathway: Prevention by Hydralazine Derivatives” by C. Camaré et al., “Cardiovascular Mitochondrial Dysfunction Induced by Cocaine: Biomarkers and Possible Beneficial Effects of Modulators of Oxidative Stress” by M. Graziani et al.), some neurological effects of modulators of oxidative stress (“Neuroprotective and Memory-Enhancing Effect of the Combined Extract of Purple Waxy Corn Cob and Pandan in Ovariectomized Rats” by W. Kirisattayakul et al. and “Alleviation of Oxidative Damage and Involvement of Nrf2-ARE Pathway in Mesodopaminergic System and Hippocampus of Status Epilepticus Rats Pretreated by Intranasal Pentoxifylline” by Y. Kang), and some effects of modulators of oxidative stress related to cell growth and cancer development (“Dihydropyridine Derivatives as Cell Growth Modulators In Vitro” by I. Bruvere et al., “A Single Zidovudine (AZT) Administration Delays Hepatic Cell Proliferation by Altering Oxidative State in the Regenerating Rat Liver” by A. Butanda-Ochoa et al., “Preclinical Antileukemia Activity Of Tramesan: A Newly Identified Bioactive Fungal Metabolite” by M. R. Ricciardi et al., and “Markers of Oxidative Stress and Inflammation in Ascites and Plasma in Patients with Platinum-Sensitive, Platinum-Resistant, and Platinum-Refractory Epithelial Ovarian Cancer” by J. C. Cantón-Romero et al.).

In particular, this special issue contains two interesting contributions coming from INSERM Toulouse. The first one is an original research paper prepared in collaboration with CNRS and Palau Sabatier University in which C. Camare et al. investigated whether 4-hydroxynonenal (4-HNE), an aldehydic lipid oxidation product abundantly present in oxidized LDL, contributes to its proangiogenic properties. Using the immunofluorescence analysis of human atherosclerotic lesions, they found colocalization of HNE adducts with CD31 (marker of the endothelium), indicating a close relationship between 4-HNE and neovascularization. Moreover, they revealed that physiological concentrations of 4-HNE also enhance the formation of tubes by human microvascular endothelial cells (HMEC-1), through mechanisms involving reactive oxygen species (ROS) and activation of the neutral type 2 sphingomyelinase and sphingosine kinase-1 (nSMase2/SK-1) pathway. Eventually, they found that carbonyl scavengers hydralazine and bisvanillyl-hydralazone inhibited such proangiogenic effects of 4-HNE. In their second paper (in this time, a review paper) prepared jointly with partners from Mexico and from INRA in Toulouse, they described in detail features of the well-known plants of *Opuntia* spp. and their possible beneficial effects on chronic diseases. Thus, M. del Socorro Santos Díaz et al. point to the differences in the phytochemical composition between wild and domesticated (*O. ficus-indica*) *Opuntia* spp., stressing that all *Opuntia* components (pear, roots, cladodes, seeds, and juice) used as nutritional and pharmaceutical agents exhibit beneficial properties mainly resulting from their high content in antioxidants, while several other phytochemical components (biopeptides, soluble fibers), which have been characterized, also contribute to the medicinal properties of *Opuntia* spp. that might have a great economic potential because these plants grow in arid and desert regions.

In the original research paper prepared by partners from Riga and from Zagreb, we found novel information about growth-modulating effects of the dihydropyridine derivatives (DHPs) with potential antioxidant capacities. Thus, I. Bruvere et al. revealed that cell-type-specific differences in the growth-modifying effects of the DHPs in vitro can be attributed only to the novel types of the DHPs, which differentiate these substances from their well-known predecessor diludine. Since the growth-modifying effects of the novel DHPs indicate possible differential effects on cancer and on nonmalignant cells, which might be also different from their antioxidant effects, these substances deserve particular attention and further studies.

The original research paper prepared by three teams from Karlsruhe, Germany, reveals the possible beneficial effects of pentaerythritol tetranitrate (PETN), which were not described before. Namely, pulmonary arterial hypertension was induced by the ranging dosage of the i.v. applied monocrotaline, which induced endothelial dysfunction, pulmonary vascular wall thickening, and fibrosis, as well as protein tyrosine nitration, thus causing an increase in pulmonary arterial pressure, followed by the increase in heart/body and lung/body weight ratios. However, in this study, S. Steven et al. found also that PETN therapy could act beneficially upon these pathophysiological processes, most likely by upregulation of heme oxygenase-1 (HO-1).
